# Pituitary Carcinoma in a Patient with an *SDHB* Mutation

**DOI:** 10.1007/s12022-017-9474-7

**Published:** 2017-03-10

**Authors:** Nicola Tufton, Federico Roncaroli, Irene Hadjidemetriou, Mary N Dang, Judit Dénes, Leonardo Guasti, Maria Thom, Michael Powell, Stephanie E Baldeweg, Naomi Fersht, Márta Korbonits

**Affiliations:** 1 Centre of Endocrinology, William Harvey Research Institute, Barts and the London School of Medicine and Dentistry, Queen Mary University of London, Charterhouse Square, London, EC1M 6BQ UK; 2 Division of Neuroscience, University of Manchester, Manchester, UK; 3 Department of Neuropathology, University College London Hospitals, WC1E 6BT, London, UK; 4 Department of Neurosurgery, University College London Hospitals, WC1E 6BT, London, UK; 5 Department of Endocrinology, University College London Hospitals, WC1E 6BT, London, UK; 6 Department of Oncology, University College London Hospitals, WC1E 6BT, London, UK

**Keywords:** Succinate dehydrogenase, *SDHB*, Paraganglioma, Pituitary carcinoma, Temozolomide

## Abstract

We present the first case of pituitary carcinoma occurring in a patient with a succinate dehydrogenase subunit B *(SDHB)* mutation and history of paraganglioma. She was initially treated for a glomus tumour with external beam radiotherapy. Twenty-five years later, she was diagnosed with a non-functioning pituitary adenoma, having developed bitemporal hemianopia. Recurrence of the pituitary lesion (Ki-67 10% and p53 overexpressed) occurred 5 years after her transsphenoidal surgery, for which she underwent two further operations followed by radiotherapy. Histology showed large cells with vacuolated clear cytoplasm with positive immunostaining for steroidogenic factor 1 (SF1) and negative staining for pituitary hormones. Four years after the pituitary radiotherapy, two metastatic deposits were identified: a foramen magnum lesion and an intradural extra-medullary cervical lesion at the level of C3/C4. There was also significant growth of the primary pituitary lesion with associated visual deterioration. A biopsy of the foramen magnum lesion, demonstrating cells with vacuolated, clear cytoplasm and positive SF1 staining confirmed a pituitary carcinoma, for which she was commenced on temozolomide chemotherapy. There was dramatic clinical improvement after three cycles and reduction in the size of the lesions was observed following six cycles of temozolomide, and further shrinkage after 10 cycles. The plan is for a total of 12 cycles of temozolomide chemotherapy. *SDH* mutation-related pituitary tumours have an aggressive phenotype which, in this case, led to metastatic disease. SF1 immunostaining was helpful to identify the tissue origin of the metastatic deposit and to confirm the pituitary carcinoma.

## Introduction

The co-occurrence of pituitary adenoma (PA) and paraganglioma is a rare event. Such association was first described in 1952 [1] but it is only recently that the underlying genetic mutations have been elucidated in some of the cases [2–5]. Mutations in one of the five succinate dehydrogenase (*SDHA-D*, *SDHA2F*) genes are most common followed by mutations in *MEN1* and *RET* genes [3–8].

Pituitary carcinoma is defined as a tumour of adenohypophyseal cells that causes cranio-spinal or systemic metastases. Pituitary carcinoma is uncommon, accounting for about 0.12% of adenohypophyseal tumours [9, 10], 6% of invasive adenomas [11], and with a reported incidence in Europe lower than 0.4 × 100,000 [12]. About 20% of pituitary carcinomas are clinically non-functioning. In such cases, unless the location of the metastatic deposits cause symptoms, patients may remain asymptomatic and the metastases are only discovered incidentally or at autopsy.

We describe the first patient with an *SDHB* mutation who developed a paraganglioma and a clinically non-functioning gonadotroph carcinoma. Metastases from the initial adenoma occurred over a decade after the first transsphenoidal surgery.

## Case Presentation

This patient presented in 1980 at the age of 28 years with unilateral deafness. She was diagnosed with a glomus tumour of the right ear based on a CT-scan and she was treated with external beam radiotherapy. At that time, her past medical and family history were otherwise unremarkable. In 2005, she developed a bitemporal hemianopia. A magnetic resonance imaging (MRI) demonstrated an intra and suprasellar lesion that had features consistent with a pituitary adenoma. She had no clinical evidence of pituitary hormone excess or deficiency, suggesting the lesion was clinically non-functioning. Urinary metanephrines were normal. She underwent transsphenoidal surgery with gross total resection of the lesion. Her visual fields fully recovered. The lesion was reported as an ‘atypical’ adenoma (WHO 2004 classification) [13] by an experienced neuropathologist but the original slides and paraffin blocks of the primary tumour were not available for review.

**Fig. 1:**
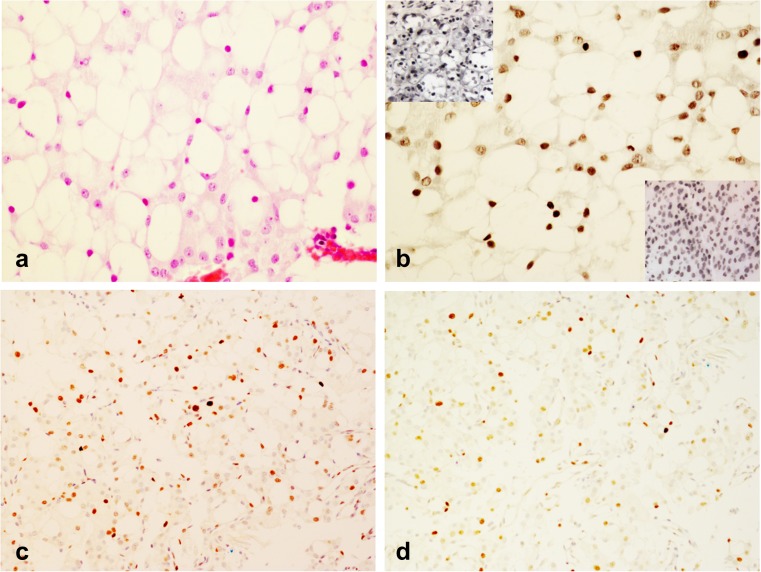
Histology of the recurrent pituitary lesion. The recurrent pituitary lesion shows acinar and lobular architecture and is composed mostly of large cells with clear, vacuolated cytoplasm reminiscent of physaliferous cells of chordoma (**a**—HE, ×40); tumour cells show nuclear expression of SF1 (**b**—immunoperoxidase, ×40, mouse anti-SF1 (sc-393,592) at dilution 1:100) top left insert shows SF1 nuclear expression in normal adrenal and lower right insert shows SF1 expression in FSH positive pituitary adenoma; up to 15% of neoplastic cells are positive for Ki-67 (**c**—immunoperoxidase, ×20, DAKO, monoclonal at dilution 1:200) and about 5% show strong nuclear expression of p53 (**d**—immunoperoxidase, ×20, DAKO, clone D07 at dilution 1:200). Ki-67 and p53 were quantified at the magnification of ×40 in the three fields showing the highest number of positive cells. The number of positive cells and the overall number of neoplastic cells was counted manually by tagging in each field, averaged and represented as a percentage of Ki-67 or p53 expressing cells against the whole number of neoplastic cells

The pituitary lesion recurred 5 years later. She underwent a second transsphenoidal operation, which was followed by transcranial debulking and fractionated rapid arc radiotherapy (50.4 Gy) a few months later. This recurrent tumour was documented in a recent study [5]. Briefly, the lesion showed acinar and lobular architecture and was composed of large cells with vacuolated, clear cytoplasm, at times mimicking physaliferous cells of chordoma (Fig. 1a). Immunostains for pituitary hormones were negative whilst neoplastic cells showed nuclear expression of steroidogenic factor 1 (SF1) (Fig. 1b) [14]. The Ki-67 labelling index was 15%, p53 was overexpressed in about 5% of tumour cells (Fig. 1c,d ) and the immunoreaction for MGMT was negative suggesting silencing of the gene. About 60% of tumour cells showed nuclear expression of MSH6. Expression of *SDHB* was faint to negative in neoplastic cells [5].

Genetic testing revealed a heterozygote pathogenic missense mutation in the *SDHB* gene (c.587G>A; p.Cys196Try, MIM 185470) [12–15].

**Fig. 2:**
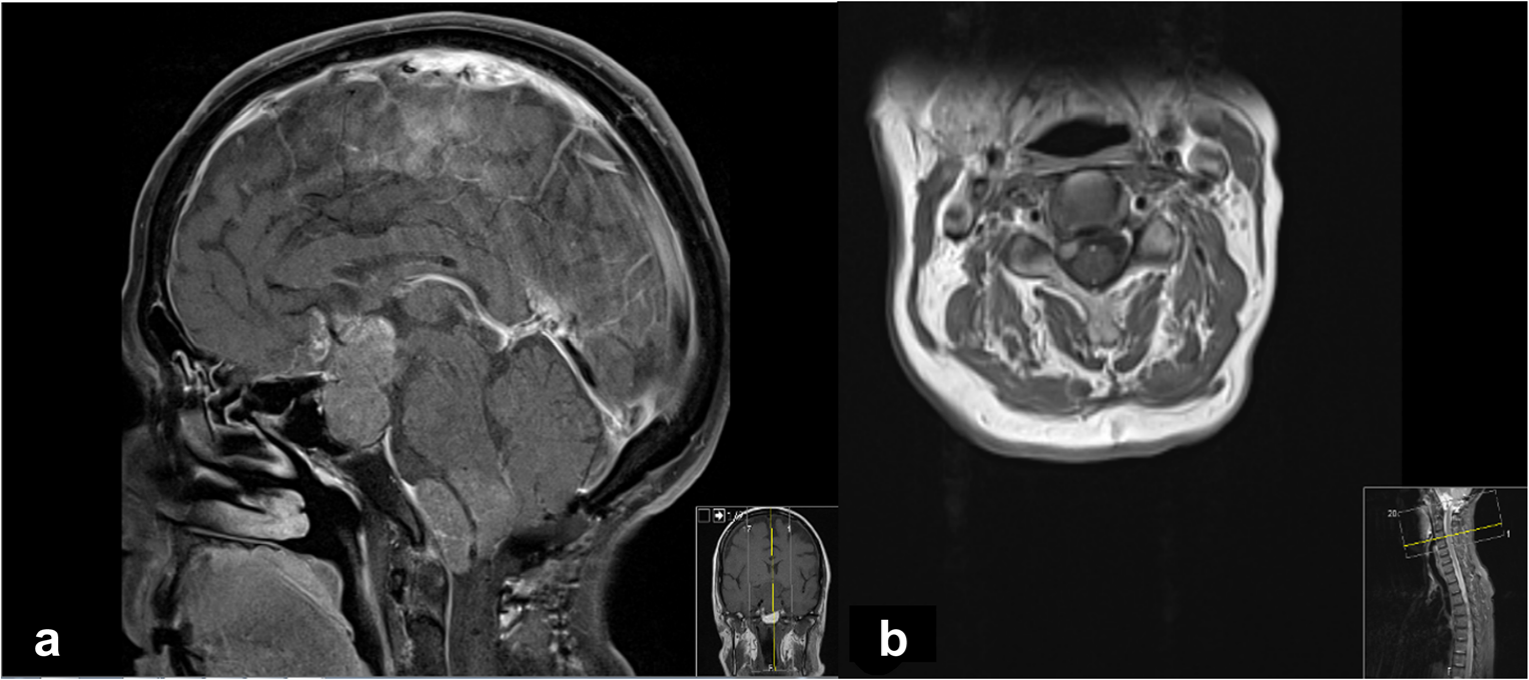
Radiological images of the pituitary lesion and metastatic deposits. **a** Sagittal section of MRI pituitary post gadolinium showing suprasellar mass and extra-axial metastatic deposit in the posterior fossa with extension into the left hypoglossal canal. **b** MRI axial sequence post gadolinium showing the intradural extra-medullary cervical metastatic deposit at the level C3/C4

The adenoma remained stable for the following 4 years. However, a surveillance MRI identified an extra-axial lesion in the foramen magnum with extension into the left hypoglossal canal (Fig. 2a). Upon review of previous MRI scans, the lesion was visible 2 years before, but grew substantially during the interval period. A further intradural extra-medullary cervical lesion was also evident at the level of C3/C4 (Fig. 2b). Both pituitary and cerebellar lesions were avid on FDG PET, but non avid on MIBG. Contrast enhancement and uptake of FDG and MIBG of the spinal lesion was conversely low.

A diagnostic biopsy from the foramen magnum lesion was taken. Following the procedure, the patient developed seizures and expressive dysphasia. A CT-scan demonstrated hydrocephalus. She improved after the insertion of a ventriculo-peritoneal shunt with some residual receptive dysphasia and right sided hemianopia. A MRI performed the following year demonstrated growth of both the suprasellar and the foramen magnum lesions, causing complete loss of vision and some distortion of the medulla. Gallium PET confirmed progressive disease and she developed Charles Bonnet syndrome (complex visual hallucinations in visually impaired patients). She was started on a 12 cycle course of temozolomide treatment (200 mg/m^2^ for 5 days, every 28 days). After three cycles she showed dramatic clinical improvement in her expressive and receptive dysphasia as well as in her visual acuity with stability on MRI. Following six and then ten temozolomide cycles, MRI demonstrated slight reduction in the size of the lesions.

**Fig. 3:**
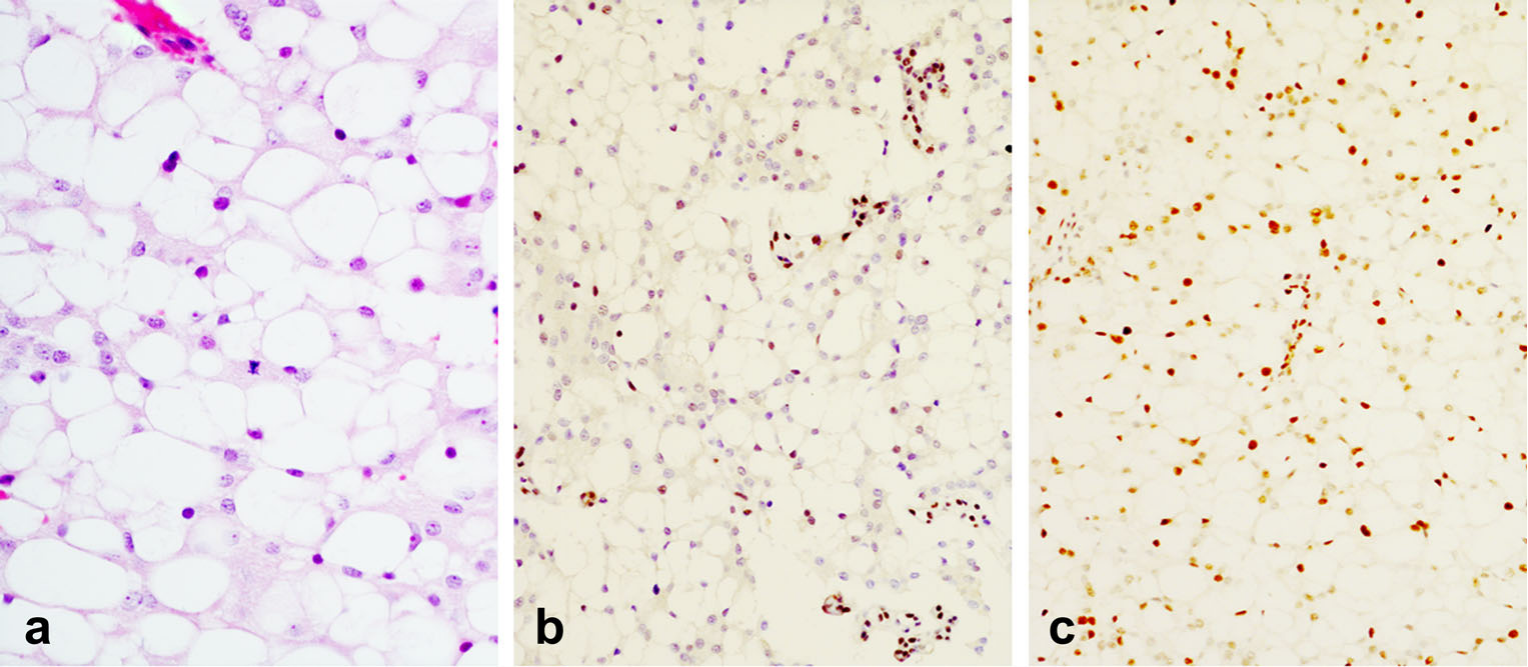
Histology of the metastatic deposit. Pathological features of the metastatic deposit are similar to the sellar recurrent tumour; neoplastic cells shows clear vacuolated cytoplasms; one mitosis is present in this field (**a**—HE, ×40); the immunoreactions for MGMT is negative in neoplastic cells; endothelial cells demonstrate normal nuclear expression (**b**—immunoperoxidase, ×20, Chemicon, monoclonal antibody at dilution 1:500); tumour cells show nuclear expression of MSH6 (**c** immunoperoxidase, ×20 using Roche clone 44 and antigen retrieval with CC1 and Optiview detection kit)

Pathological examination of the metastatic deposit demonstrated features similar to those of the recurrent pituitary adenoma. The neoplastic cells had the same degree of vacuolisation. Mitotic count was lower than 1 × 10 high power field. The neoplastic cells were positive for cytokeratin MNF116, cytokeratin CAM5.2, synaptophysin and the pituitary transcription factor SF1, and they were negative for pituitary hormones, TTF-1, EMA, S-100 protein, inhibin, GFAP, brachyury, common α-subunit and oestrogen receptor. Ki-67 expression was 10% and about 4–5% showed intense nuclear expression of p 53. Similar to the sellar recurrence, tumour cells were negative for MGMT and about 70% expressed MSH6 (Fig. 3).

We note that in 2007, the patient’s sister at the age of 51 years developed an anaplastic oligodendroglioma (WHO grade III) and died of the disease in the same year. Immunostaining for SDHB showed normal expression in the tumour cells.

## Discussion

We have documented the first case of pituitary carcinoma occurring in a patient with an *SDH* mutation. Pituitary carcinomas rarely occur in genetic syndromes; examples of pituitary carcinoma patients with *MEN1* mutations have been documented [15–17], whilst *AIP*, *PRKAR1A*, *DICER1* and *GPR101* mutations have not been associated with metastatic spread to date.

Pituitary adenomas occurring in patients with *SDH* mutations or adenomas bearing somatic mutations are more often lactotroph adenomas followed by gonadotroph adenomas and somatotrophinomas [5].

Vacuolated, clear cytoplasm was the hallmark of the pituitary tumour and posterior fossa metastasis and the fact that it was present in both lesions indicates that vacuoles are genuine features of the tumour rather than an artefact. Our previous work documented vacuolated cytoplasm in *SDH*-mutated adenomas but not in patients with pituitary adenomas and phaeochromocytomas due to *MEN1* or *VHL* genes. This evidence suggests that vacuoles are due to inactivation of the SDH complex but the mechanism leading to their formation remains unclear. In our experience, vacuoles range from small and multiple to large, occupying most of the cytoplasm and mimic signet-ring cells of metastatic adenocarcinoma, clear cells of renal cell carcinoma or physalipherous cells of chordoma. In an *Sdhb* (+/−) animal model, giant mitochrondria were shown in the pituitary tumours, which may account for the cytoplasmic vacuoles [4], but we were unable to show positive mitochrondrial markers in human cases in our previous study [5].

Although not limited to SDH-related pituitary tumours, widespread clear cell changes in pituitary samples should alert the pathologists and prompt further investigations. Immunostaining with SDHA and SDHB antibodies is a valuable diagnostic tool to screen adenomas prior to requesting genetic analysis. Gill et al. [8] investigated a large cohort of adenomas and identified 1/309 patient whose adenoma was negative for SDHA and SDHB. Genetic analysis revealed two somatic inactivating mutations in *SDHA* in this single case*.*

The primary and recurrent adenohypophyseal tumour in our patient had features of an atypical adenoma. Initially defined as adenomas with uncertain malignant potential, the definition of atypical adenoma remains controversial. Two recent studies have attempted to provide more reproducible criteria for predicting the outcome of pituitary adenomas. Mitotic activity, increased Ki-67 labelling index and overexpression of the oncoprotein p53 remain helpful for a diagnosis of adenoma with high risk of recurrence. Trouillas et al. [18] proposed a staging system where pathological criteria are integrated with extension and invasion to the surrounding structures. The pituitary tumour sample from the second transsphenoidal operation from this patient (4 years before diagnosis of metastatic disease) would belong to the 2b group of the Trouillas classification. A more recent study based on data from the German Pituitary Adenoma Registry suggested cut off values for Ki-67 equal to or >4% and p53 equal to or >2% and indicated that these two markers are more reliable and reproducible than mitotic count for the diagnosis of high-risk adenoma [19].

Metastatic deposits in our patient occurred almost a decade after the diagnosis of PA. Studies have attempted a distinction between pituitary carcinomas that show rapidly aggressive course and those showing a more indolent behaviour [20, 21]. These latter lesions account for the majority of cases and their behaviour is similar to invasive adenoma except for presence of metastases. Patients’ survival can be long with reports documenting up to 25 years. In contrast, those presenting as a malignant appearing lesion show multiple recurrences in a short time from onset, metastatic spread occurs early and have rapidly fatal progression with a survival that is often shorter than 1 year [20]. The underlying genetic makeup of the two groups is unknown.

We think it is unlikely that the initial radiotherapy (RT) for the paraganglioma caused the pituitary tumour, or contributed to its progression, as the pituitary fossa was well outside the fields of irradiation. Our patient had an excellent clinical response to temozolomide and her disease burden is now stable, having completed ten of the planned 12 cycles of temozolomide since the discovery of the two metastatic deposits. Following the original observation of positive response of pituitary tumours to temozolomide [22], several studies have advocated the use of this drug to control invasive adenomas and carcinomas. Overall, the benefit of temozolomide is modest but still significant given the natural history of pituitary carcinoma [10, 22–26]. Haematological toxicity is a complication that may require dose reduction and seldom withdrawal of the agent. Low-level MGMT immunoexpression was correlated with a favourable response [27, 28]. However, no constant association was found between *MGMT* promoter methylation and response [28, 29]. In a recent study, 10/13 (77%) temozolomide-treated ‘atypical’ adenomas or carcinomas responded, either completely or partially [30]. Six cases (46%) recurred after initial response. MGMT immunoexpression had no relation with the response to temozolomide, whilst immunoexpression of MSH6 was proven to correlate significantly with the response [30–32]. In the mismatch repair pathway, base mismatches are detected by the heterodimers of MSH2 and MSH6, which assist another heterodimeric complex of MLH1 and PMS2 [33]. Mismatch repair pathway stimulates DNA damage-induced G2 checkpoint and apoptosis during DNA synthesis. It was suggested that the inactivation of MMR is associated with tolerance to the cytotoxic effects of alkylating agents [34, 35].

In conclusion, we have documented the first pituitary carcinoma in a patient with a germline mutation of the *SDHB* gene and paraganglioma. Differential diagnosis between pituitary carcinoma and other clear cell tumours in our patient was challenging given the unusual features of the lesion. Immunostaining for the SF1 was helpful to identify the tissue origin of the metastatic deposit.

## Abbreviations

*SDHB*: Succinate dehydrogenase subunit B
SF1: Steroidogenic factor 1
MGMT: O^6^ methyl guanine methytransferase
MSH6: DNA mismatch repair protein
MEN: Multiple endocrine neoplasia
VHL: von Hippel Lindau
